# S-acylation and membrane localization of the small GTPase ARL15 are mediated by the Golgi S-acyltransferases ZDHHC7 and ZDHHC3

**DOI:** 10.1016/j.jbc.2026.111460

**Published:** 2026-04-16

**Authors:** Takeshi Chino, Makoto Araki, Yuki Ashi, Yusuke Izawa, Yuji Nunami, Yosuke Ichikawa, Itsuki Kuroiwa, Kenji Kontani

**Affiliations:** Department of Biochemistry, Meiji Pharmaceutical University, Tokyo, Japan

**Keywords:** ARL15, golgi apparatus, small GTPases, S-acylation, palmitoylation, ZDHHC

## Abstract

ARL15 is a member of the ARF-like (ARL) family of small GTPases, implicated in the regulation of ion homeostasis and metabolic signaling pathways. Although ARL15 has been suggested to undergo S-acylation—a reversible lipid modification that governs membrane association and trafficking—the stoichiometry of this modification and the responsible S-acyltransferases have remained unclear. Here, we systematically characterized the S-acylation of ARL15 and identified the enzymes mediating this modification. Using acyl-PEGyl exchange gel-shift assays, we show that ARL15 is triply S-acylated at three conserved N-terminal cysteine residues (Cys17, Cys22, and Cys23) in HEK293T cells. Single cysteine-to-serine mutations substantially reduced S-acylation, whereas substitution of all three cysteines abolished it entirely. Loss of S-acylation disrupted membrane association of ARL15, as shown by confocal imaging and subcellular fractionation. A candidate screen using siRNA knockdown and CRISPR/Cas9-mediated gene disruption revealed that the Golgi-localized S-acyltransferases ZDHHC7 and ZDHHC3 mediate ARL15 S-acylation in a partially redundant or parallel manner. Dual inhibition of both enzymes led to a marked reduction in S-acylation and redistributed ARL15 from membranes to the cytosol. These findings elucidate the stoichiometry and enzymatic regulation of ARL15 S-acylation and provide mechanistic insight into its subcellular localization.

Small guanosine triphosphate (GTP)-binding proteins (Small GTPases) consist of more than 150 members in humans and regulate a wide variety of cellular responses, including cell proliferation and differentiation. Small GTPases are classified into five subfamilies—RAS, RHO/RAC, RAB, ARF/ARL and RAN—based on their sequence similarities and functions (1, 2). The RAS subfamily is extensively studied due to its involvement in oncogenesis and cancer progression. Members of this subfamily, such as KRAS, HRAS, and NRAS, are frequently mutated in human cancers, driving aberrant signaling cascades that promote tumorigenesis. The RHO/RAC subfamily regulates actin cytoskeleton dynamics and cell migration, influencing processes crucial for embryonic development and tissue morphogenesis. Additionally, the RAB subfamily governs intracellular vesicle trafficking, ensuring the precise targeting and fusion of vesicles with their respective cellular compartments.

The ARF/ARL subfamily includes ARFs, ARLs (ARF-like), and SARs. ARF GTPases (five members in humans) regulate membrane traffic mainly at the Golgi: they coordinate vesicle budding and cargo transport between the Golgi apparatus and other cellular compartments, ensuring proper protein sorting and secretion. The activity of ARF GTPases is tightly regulated by guanine-nucleotide exchange factors (GEFs) and GTPase-activating proteins (GAPs). GEFs promote the exchange of guanosine diphosphate (GDP) for GTP, thereby activating ARF GTPases and facilitating their involvement in membrane trafficking events. Conversely, GAPs catalyze the hydrolysis of GTP to GDP, leading to the inactivation of ARF GTPases and the termination of their signaling functions, thus ensuring precise spatiotemporal control over membrane traffic dynamics at the Golgi apparatus (3, 4, 5).

Compared to ARFs, ARL GTPases (21 members in humans) display more diverse cellular functions and are involved in membrane traffic, microtubule organization, mitochondrial dynamics, and primary cilia assembly; however, their regulatory factors (*i.e.*, GEFs and GAPs) remain less defined. ARL15 is an ARL-family member identified by homology to ARFs. Genome-wide association studies have implicated ARL15 in several traits and diseases, including diabetes (6, 7, 8, 9, 10). Recent studies have indicated that ARL15 is involved in intracellular Mg^2+^ homeostasis *via* interaction with Mg^2+^ transporter CNNMs and cation channel TRPM7 (11, 12, 13). ARL15 has been suggested to be localized to the Golgi/trans-Golgi network (TGN) and the plasma membrane, and S-acylation (palmitoylation) has been proposed to contribute to its membrane association (14). However, the stoichiometry of ARL15 S-acylation and the responsible enzymes for the S-acylation remained unclear. In the present study, we investigated ARL15 S-acylation and found that the majority of intracellular ARL15 is S-acylated at all three N-terminal Cys residues. Each of the Cys residues is essential for maintaining efficient S-acylation; substitution of any single Cys residue to Ser drastically reduces ARL15 S-acylation. We also found that the Golgi-localized S-acyltransferases zinc-finger DHHC (ZDHHC)7 and ZDHHC3 mediate the S-acylation and the membrane localization of ARL15.

## Results

### ARL15 is S-acylated at three N-terminal cysteine residues

**Figure 1:**
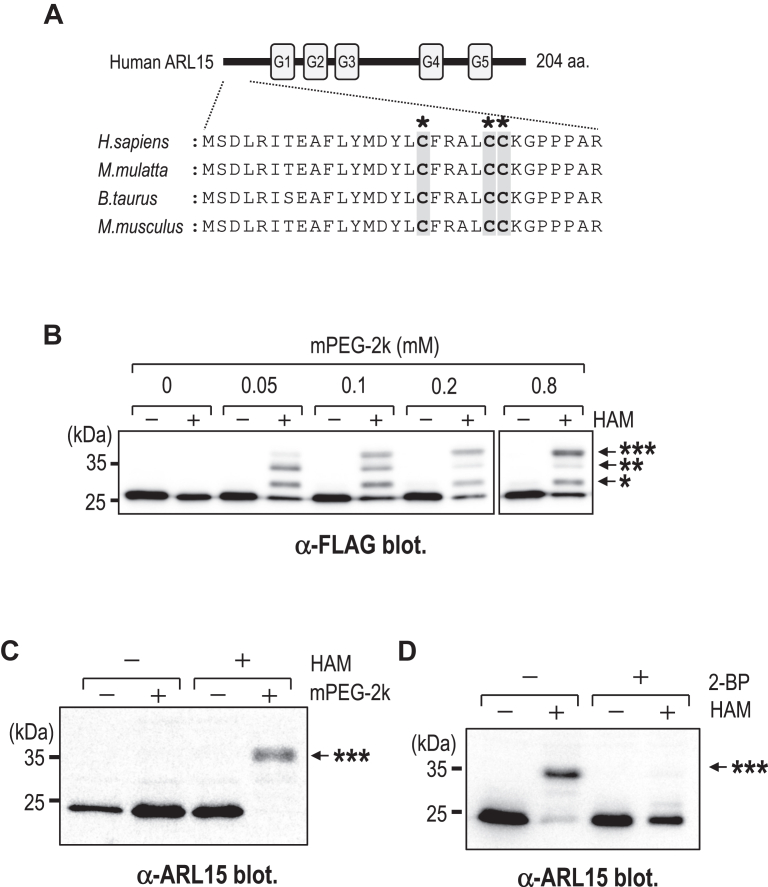
**ARL15 is S-acylated at three conserved N-terminal cysteine residues.***A*, sequence alignment of the N-terminal region of ARL15 among representative mammals. Three conserved cysteine residues (Cys17, Cys22, and Cys23 in human) are highlighted. *B*, APEGS assay of ARL15-FLAG expressed in HEK293T cells under various concentrations of mPEG-2k with (+) or without (−) HAM, as indicated. Bands marked with ∗∗∗, ∗∗, and ∗ represent putative tri-, di-, and mono-S-acylated forms, respectively. *C*. APEGS assay of endogenous ARL15 in HEK293T cells. A band marked with ∗∗∗ represents a putative tri-S-acylated form of ARL15. *D*, effect of 2-bromopalmitate (2-BP) on ARL15 S-acylation. HEK293T cells were cultured for ∼ 16 h in the absence (−) or presence (+) of 100 μM 2-BP and subjected to APEGS assay (±HAM, as indicated). A band marked with ∗∗∗ represents a putative tri-S-acylated form of ARL15. HAM, hydroxylamine; mPEG-2k, methoxy-PEG-maleimide (2 kDa); 2-BP, 2-bromopalmitate.

The membrane association of most ARF GTPases is mediated by an N-terminal amphipathic α-helix and an N-myristoyl group attached to the second glycine residue (15). In the case of ARL15, however, the second amino acid is a serine, indicating that ARL15 is unlikely to undergo myristoylation. Instead, ARL15 possesses three highly conserved cysteine residues (Cys17, Cys22, and Cys23 in human ARL15) (Fig. 1*A*), which are potential S-acylation sites. We therefore examined the S-acylation status of ARL15 using the acyl-PEGyl exchange gel-shift (APEGS) assay, a method that enables detection and estimation of protein S-acylation levels and stoichiometry (16, 17, 18).

To optimize assay conditions, we expressed ARL15-FLAG in HEK293T cells and treated the lysates with increasing concentrations of mPEG-2k (a methoxy-PEG-maleimide with a 2000 Da PEG chain) (Fig. 1*B*). At 0.1 mM mPEG-2k, three discrete shifted bands (marked by asterisks) were observed. As the mPEG-2k concentration increased, the band with the lowest electrophoretic mobility—presumably corresponding to triply S-acylated ARL15-FLAG—became the predominant species. We next analyzed endogenous ARL15 S-acylation under optimized APEGS conditions (4 mM mPEG-2k), which revealed a prominent band corresponding to the putative triply S-acylated forms (Fig. 1*C*). This band was abolished by treatment with 2-bromopalmitate (2-BP), a broad-spectrum inhibitor of protein S-acylation (palmitoylation) (Fig. 1*D*). Together, these findings indicate that in HEK293T cells, ARL15 is predominantly S-acylated at all three N-terminal cysteine residues.

### Mutation of each cysteine residue impairs ARL15 S-acylation

**Figure 2:**
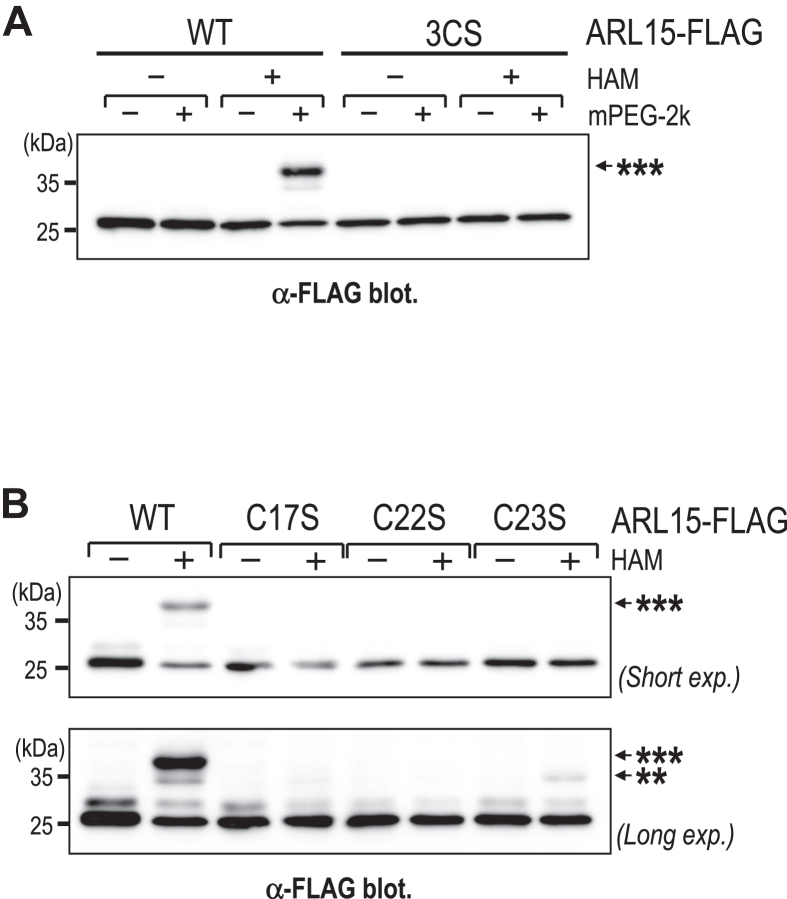
**Mutation of individual cysteine residues reduces ARL15 S-acylation.***A*, APEGS assay of ARL15-FLAG WT and the triple cysteine-to-serine mutant (3CS), in which all three cysteine residues (Cys17, Cys22, and Cys23) were substituted with serines. A band marked with ∗∗∗ represents a putative tri-S-acylated form of ARL15. *B*, APEGS assay of ARL15-FLAG WT and single cysteine-to-serine mutants (C17S, C22S, C23S). Bands marked with ∗∗∗ and ∗∗ represent putative tri- and di-S-acylated forms of ARL15, respectively. Upper panel: short exposure; lower panel: long exposure of the same blot. mPEG-2k, methoxy-PEG-maleimide (2 kDa).

To assess the contribution of the three N-terminal cysteine residues (Cys17, Cys22, and Cys23) to ARL15 S-acylation, we performed APEGS assays using various cysteine-to-serine mutants. WT ARL15-FLAG (ARL15/WT-FLAG) was predominantly triply S-acylated, whereas the triple mutant ARL15/3CS completely lacked S-acylation (Fig. 2*A*). Each single mutant (ARL15/C17S, C22S, and C23S) showed a marked reduction, indicating that all three cysteines contribute to efficient modification (Fig. 2*B*). Notably, a minor fraction of ARL15/C23S retained S-acylation, likely at the remaining two sites (Fig. 2*B*, *lower panel*). These findings collectively highlight the essential role of each individual cysteine residue in enabling full S-acylation of ARL15.

### Intracellular distribution of ARL15-FLAG in HEK293T cells

**Figure 3:**
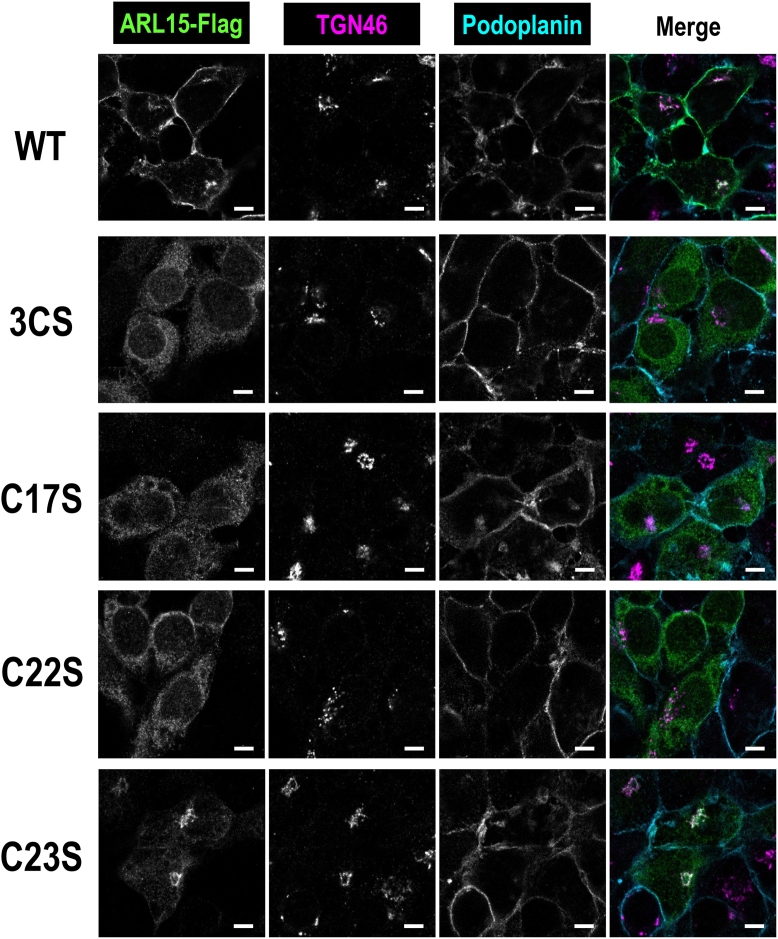
**Intracellular localization of ARL15-FLAG WT and its cysteine-to-serine mutants.** Confocal images of HEK293T cells expressing ARL15-FLAG WT or its cysteine-to-serine mutants. The cells were coimmunostained with antibodies against the FLAG-tag, TGN46 (trans-Golgi network marker), and podoplanin (plasma membrane marker). The scale bar repreesents, 5 μm.

**Figure 4:**
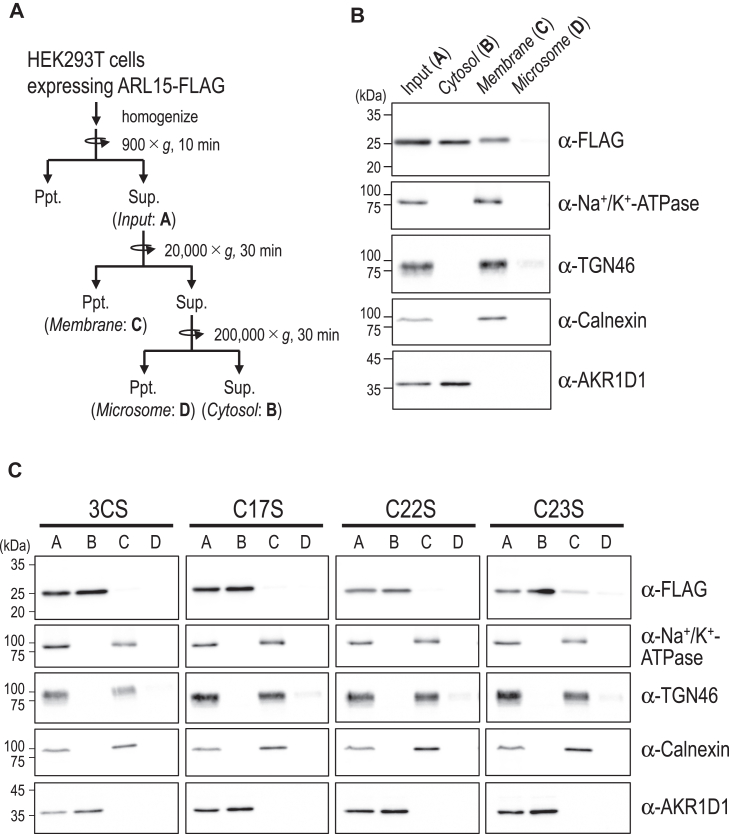
**Subcellular fractionation of ARL15-FLAG WT and its cysteine-to-serine mutants.***A*, schematic overview of the cell fractionation protocol used to separate cytosolic, membrane, and microsomal fractions. HEK293T cells expressing ARL15-FLAG were homogenized and subjected to sequential centrifugation: 900*g* for 10 min to remove nuclei and debris, 20,000*g* for 30 min to obtain cytosolic and membrane fractions, and 200,000*g* for 30 min to separate microsomal and soluble fractions. *B* and *C,* immunoblot analysis of subcellular fractions prepared from HEK293T cells expressing ARL15-FLAG WT (*B*) or its mutants (*C*). ARL15-FLAG and organelle marker proteins (Na^+^/K^+^-ATPase, plasma membrane; TGN46, trans-Golgi network; calnexin, endoplasmic reticulum; AKR1D1, cytosol) were detected using the indicated antibodies.

We next examined the subcellular localization of ARL15-FLAG by indirect immunofluorescence (Fig. 3). ARL15/WT-FLAG localized predominantly to the plasma membrane and the TGN, with weaker cytosolic signal, whereas ARL15/3CS, C17S, and C22S were largely cytosolic. ARL15/C23S localized to the cytoplasm and the TGN but was largely absent from the plasma membrane. To further probe membrane association, we performed subcellular fractionation of HEK293T cells: post-nuclear supernatants were fractionated by sequential centrifugation into cytosolic, membrane, and microsomal fractions (Fig. 4*A*). In agreement with Figure 3, ARL15/WT-FLAG was detected in both membrane and cytosolic fractions (Fig. 4*B*). Notably, endogenous ARL15 is predominantly S-acylated (Fig. 1*C*) and is recovered mainly in the membrane fraction under control conditions (Fig. 8*B*; see below). Although ectopically expressed ARL15-FLAG undergoes S-acylation, a substantial fraction remains non-S-acylated, likely due to overexpression (Fig. 2*A*). We therefore interpret the cytosolic pool of ARL15/WT-FLAG in the fractionation assay as representing, at least in part, the non-S-acylated population. In contrast to the WT, the ARL15/3CS, C17S, and C22S mutants were predominantly cytosolic; the ARL15/C23S mutant retained mostly cytosolic with a minor membrane-associated fraction (Fig. 4, *B* and *C*). These findings indicate that S-acylation at the three N-terminal cysteines is important for efficient membrane localization of ARL15.

**Figure 5:**
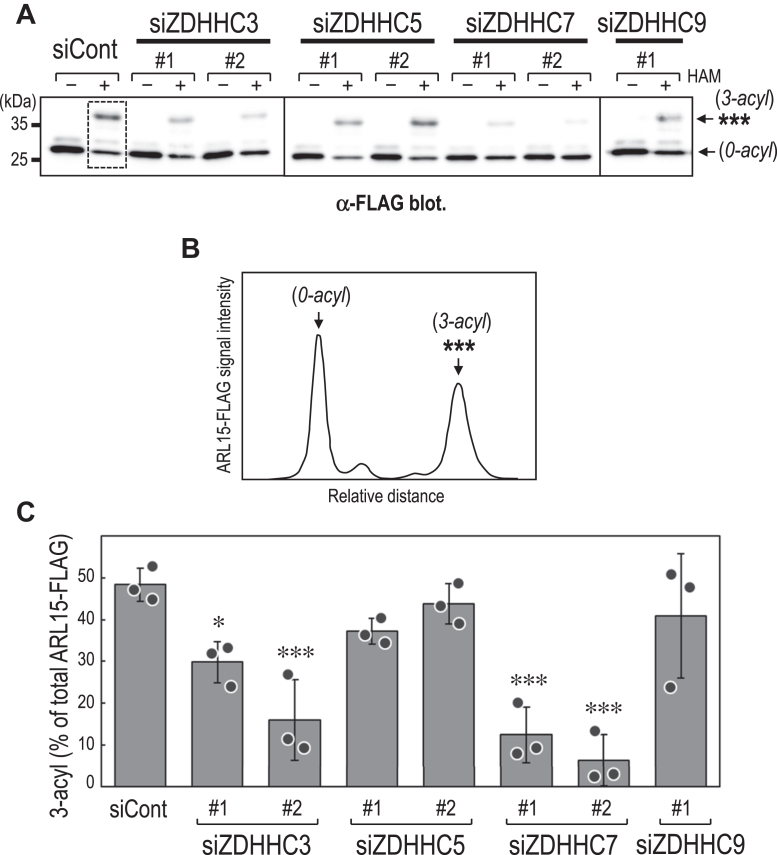
**Effects of knockdown of ZDHHC-family enzymes on ARL15 S-acylation.***A*, APEGS assay of ARL15-FLAG expressed in HEK293T cells treated with control siRNA (*siCont*) or siRNAs targeting individual ZDHHC family members (*siZDHHC3, siZDHHC5, siZDHHC7, or siZDHHC9*). The band marked with ∗∗∗ represents a putative tri-S-acylated form of ARL15 (hereafter, *3-acyl*). The band labeled *0-acyl* (*arrow*) represents the non-S-acylated form of ARL15-FLAG. *B*, representative lane profile of ARL15-FLAG signals from the APEGS assay. Signal intensity within the *dashed rectangular box* in *panelA* was quantified using ImageJ (https://imagej.net/ij/). The x-axis represents relative migration distance (gel mobility), and the y-axis indicates ARL15-FLAG signal intensity. *C*, quantification of the putative tri-S-acylated ARL15-FLAG species in cells treated with control or ZDHHC-targeting siRNAs. For each sample, the peak area corresponding to the 3-acyl band was calculated from the lane profile and expressed as a percentage of the total ARL15-FLAG signal. Data are mean ± SD from three independent experiments; individual data points are shown. ∗*p*< 0.05, ∗∗∗*p*< 0.001 (one-way ANOVA with Dunnett’s *post hoc* test *versus* siCont). HAM, hydroxylamine; siCont, control siRNA.

**Figure 6:**
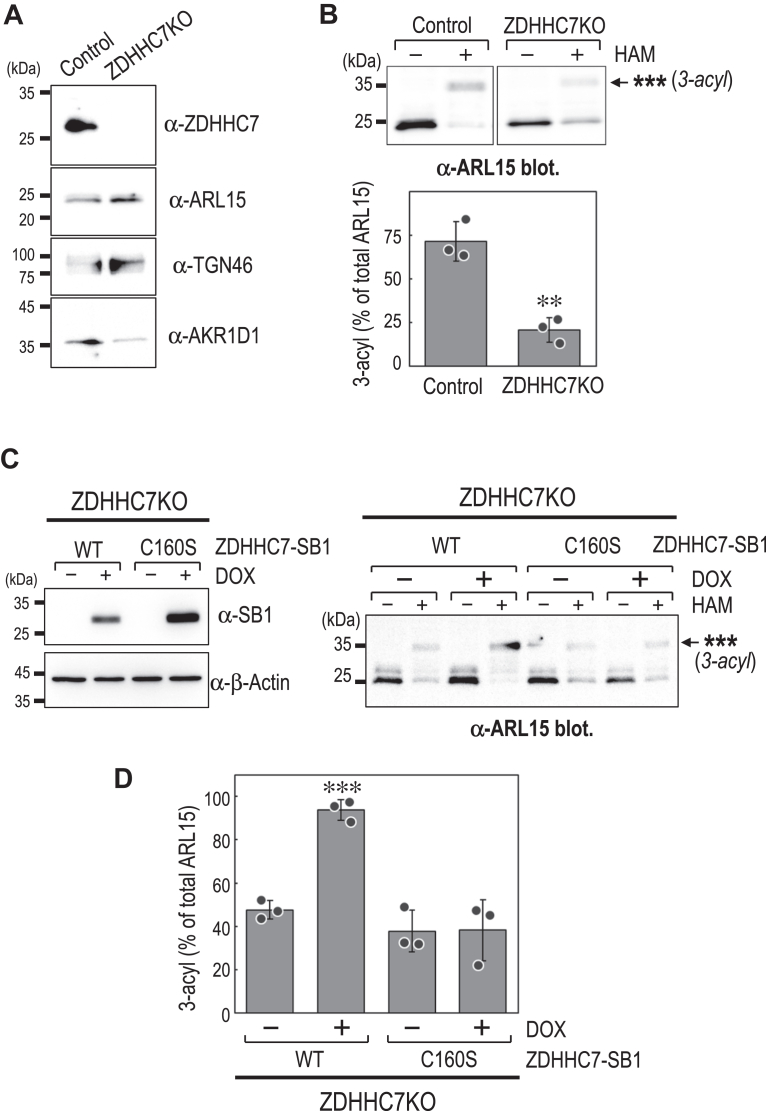
**ZDHHC7 is essential for ARL15 S-acylation.***A*, immunoblot confirming the loss of ZDHHC7 protein in ZDHHC7-KO (ZDHHC7KO) HEK293T cells. Whole-cell lysates from control and ZDHHC7KO HEK293T cells were analyzed by Western blot using the indicated antibodies. *B*, APEGS assay of endogenous ARL15 in control and ZDHHC7KO HEK293T cells (*upper panel*). Bands marked with ∗∗∗ represent a putative tri-S-acylated form of ARL15. Quantification of the 3-acyl ARL15 band, expressed as a percentage of the total ARL15 signal (*lower panel*). Data are mean ± SD from three independent experiments; individual data points are shown. ∗∗*p*< 0.01 (unpaired two-tailed Student’s *t* test). *C*, immunoblot analysis verifying the expression of SB1-tagged ZDHHC7 (WT or catalytically inactive mutant C160S) in ZDHHC7KO HEK293T cells (*left panel*). Blots were probed with the indicated antibodies. APEGS assay of endogenous ARL15 in ZDHHC7KO HEK293T cells expressing SB1-tagged ZDHHC7 WT or C160S mutant (*right panel*). Bands marked with ∗∗∗ represent a putative tri-S-acylated form of ARL15. *D*, quantification of the 3-acyl ARL15 band shown in (*C*), expressed as a percentage of the total ARL15 signal. Data are mean ± SD from three independent experiments; individual data points are shown. ∗∗∗*p*< 0.001 (unpaired two-tailed Student’s *t* test). HAM, hydroxylamine; SB1, SB1 tag; DOX, doxycycline.

**Figure 7:**
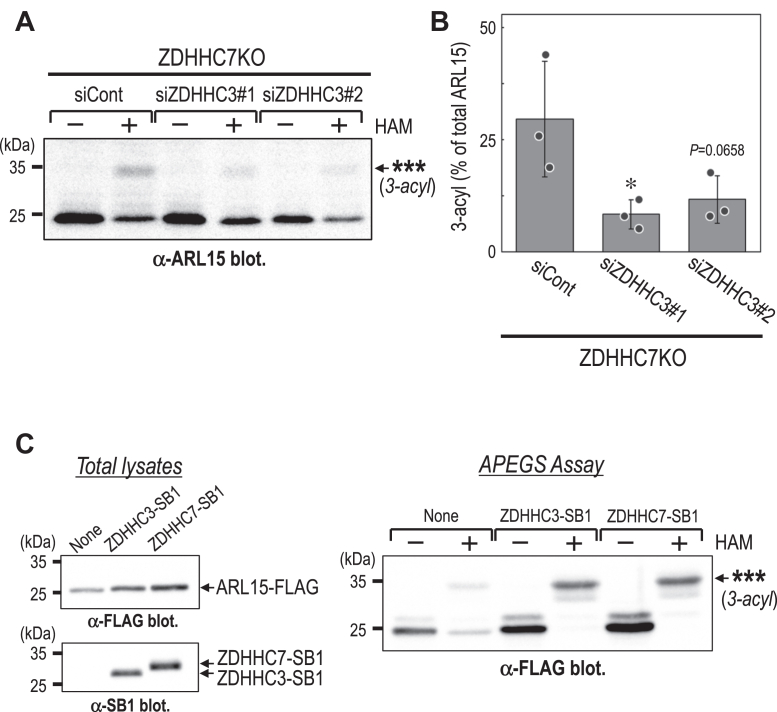
**ZDHHC7 and ZDHHC3 mediate ARL15 S-acylation.***A*, APEGS assay of ARL15 in ZDHHC7KO HEK293T cells treated with control siRNA (*siCont*) or siRNAs targeting ZDHHC3 (*siZDHHC3*). Bands marked with ∗∗∗ represent a putative tri-S-acylated form of ARL15. *B*, quantification of the 3-acyl ARL15 band shown in (*A*). Data are mean ± SD from three independent experiments; individual data points are shown. ∗*p*< 0.05 (one-way ANOVA with Dunnett’s *post hoc* test *versu*s siCont). *C*, cell lysates from HEK293T cells expressing ARL15-FLAG alone or co-xpressing ZDHHC3-SB1 or ZDHHC7-SB1 were subjected to Western blot analysis (*left*) and APEGS assay (*right*). Blots were probed with the indicated antibodies. Bands marked with ∗∗∗ represent a putative tri-S-acylated form of ARL15. HAM, hydroxylamine; siCont, control siRNA; SB1, SB1 tag.

**Figure 8:**
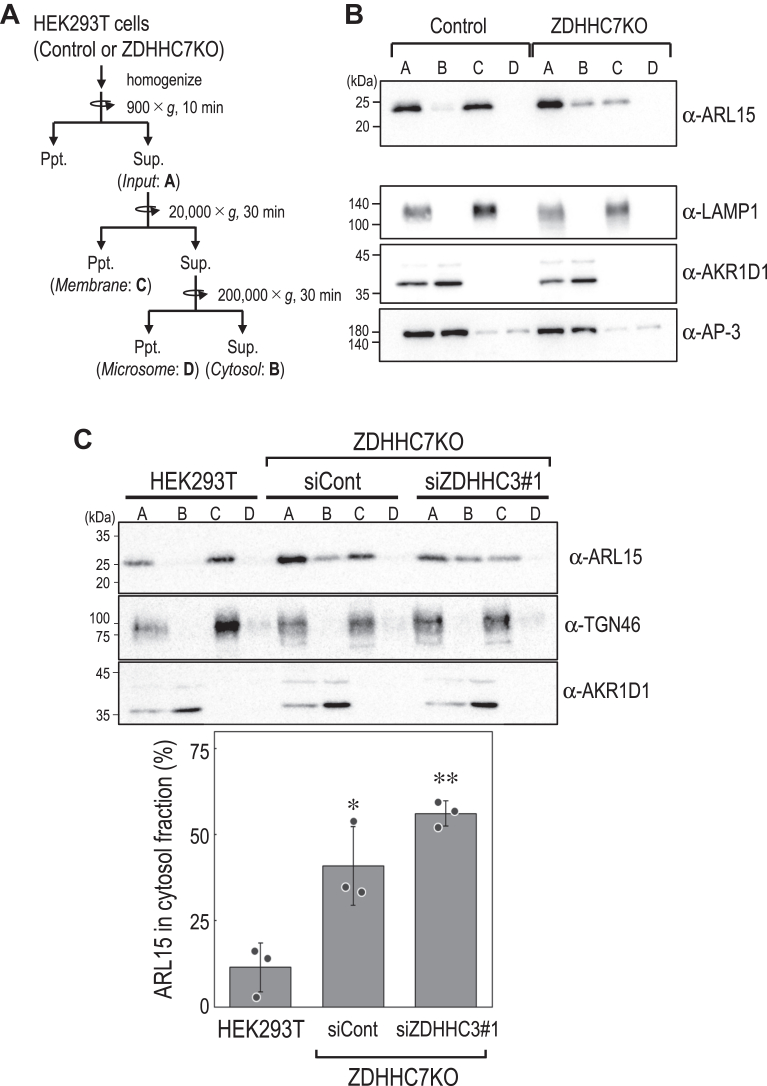
**ARL15 membrane association requires ZDHHC7 and ZDHHC3.***A*, schematic diagram of the subcellular fractionation protocol of control and ZDHHC7KO HEK293T cells (as shown in Fig. 4*A*). *B*, immunoblot analysis of subcellular fractions prepared from control and ZDHHC7KO HEK293T cells. Endogenous ARL15 and organelle marker proteins (LAMP1, late endosomes/lysosomes; AKR1D1, cytosol; AP-3, transport vesicles) were detected using the indicated antibodies. *C*, immunoblot analysis of subcellular fractions prepared from control and ZDHHC7KO HEK293T cells treated with control siRNA (*siCont*) or siRNAs targeting ZDHHC3 (*siZDHHC3*) (*upper panel*). Endogenous ARL15 and organelle marker proteins (TGN46, trans-Golgi network; AKR1D1, cytosol) were detected using the indicated antibodies. Quantification of cytosolic ARL15 levels, based on ImageJ analysis of ARL15 band intensities, expressed as a percentage of the estimated total cellular ARL15 levels (*lower panel*). Data are mean ± SD from three independent experiments; individual data points are shown. ∗*p*< 0.05, ∗∗*p*< 0.01 (one-way ANOVA with Dunnett’s *post hoc* test *versus* WT HEK293T cells). siCont, control siRNA

### Effect of siRNA-mediated knockdown of ZDHHC-family members on ARL15 S-acylation

Protein S-acylation is catalyzed by a family of S-acyltransferases containing a conserved ZDHHC domain (19, 20, 21). To identify the relevant enzymes for ARL15, we examined siRNA-mediated knockdown of selected ZDHHCs. Among the 23 known human ZDHHCs, we focused on ZDHHC3 and ZDHHC7, which are cis/medial Golgi-localized enzymes that have been implicated in S-acylation of diverse substrates, including soluble/peripheral proteins, and on ZDHHC5 and ZDHHC9, which localize predominantly to the plasma membrane and the early secretory pathway/Golgi region (often described as including the TGN), respectively (22, 23). The efficiency of siRNA knockdown was validated by quantitative PCR (*SI Appendix*, Fig. S1). We then performed APEGS assays on lysates from HEK293T cells transfected with control or ZDHHC-targeting siRNAs (Fig. 5*A*) and quantified the putative triply S-acylated ARL15-FLAG (3-acyl) as a percentage of total ARL15-FLAG signal (Fig. 5, *B* and *C*). Knockdown of ZDHHC7 markedly reduced ARL15 S-acylation, ZDHHC3 caused a moderate reduction, whereas ZDHHC5 and ZDHHC9 had little to no discernible effect, suggesting that ZDHHC7, and to a lesser extent ZDHHC3, are the principal contributors in HEK293T cells.

### ZDHHC7 and ZDHHC3 mediate ARL15 S-acylation and membrane association

The substantial reduction in ARL15 S-acylation upon ZDHHC7 and ZDHHC3 knockdown prompted further analysis. We generated ZDHHC7-KO (ZDHHC7KO) cells (Fig. 6*A*), which showed a marked decrease in ARL15 S-acylation (Fig. 6*B*), consistent with the siRNA data. This reduction was rescued by ectopic expression of WT ZDHHC7-SB1, but not by the catalytically inactive mutant ZDHHC7(C160S)-SB1 (24, 25), confirming that the enzymatic activity of ZDHHC7 is required for ARL15 S-acylation (Fig. 6, *C* and *D*).

To determine whether ZDHHC3 contributes in the absence of ZDHHC7, we performed siRNA-mediated knockdown of ZDHHC3 in ZDHHC7KO cells. Under these conditions, ARL15 S-acylation was further reduced compared with ZDHHC7KO alone (Fig. 7, *A* and *B*), indicating that ZDHHC3 contributes to ARL15 S-acylation when ZDHHC7 is absent. These findings suggest that ZDHHC7 and ZDHHC3 act in a partially redundant and/or parallel manner.

To further validate their roles, we examined the effect of overexpressing these enzymes. Coexpression of either ZDHHC7-SB1 or ZDHHC3-SB1 with ARL15-FLAG resulted in a pronounced increase in ARL15 S-acylation, with the near-complete disappearance of the non-S-acylated ARL15 band observed when ARL15-FLAG was expressed alone (Fig. 7*C*), confirming that both enzymes can promote ARL15 S-acylation.

We next assessed membrane association. Subcellular fractionation revealed that endogenous ARL15 was predominantly recovered in the membrane fraction in control cells, whereas ZDHHC7KO increased the cytosolic pool, which was further elevated by ZDHHC3 knockdown (Fig. 8, *A*–*C*). Thus, ZDHHC7 and ZDHHC3 are required not only for ARL15 S-acylation but also for its membrane association. Notably, the reduction of ARL15 in the membrane fraction upon ZDHHC7KO was not accompanied by a commensurate increase in the cytosolic fraction (Fig. 8*B*), suggesting that loss of S-acylation may decrease the steady-state abundance of ARL15. Consistent with this idea, overexpression of ZDHHC3/7 increased apparent ARL15 levels (Fig. 7*C*), raising the possibility that S-acylation stabilizes ARL15 and/or protects it from degradation.

## Discussion

In this study, we investigated the S-acylation of ARL15 in HEK293T cells and showed that ARL15 is modified at three conserved cysteine residues located near its N-terminus. Both immunofluorescence staining and subcellular fractionation analyses revealed that this S-acylation is essential for the proper membrane localization of ARL15. Furthermore, we identified the Golgi-localized S-acyltransferases ZDHHC7 and ZDHHC3 as key enzymes responsible for mediating both the S-acylation and membrane association of ARL15.

While previous studies and S-palmitoylation databases have suggested that ARL15 is subject to S-acylation, the precise stoichiometry and number of modification sites have remained unclear. Our data showed that, in HEK293T cells, ARL15 is predominantly S-acylated at all three N-terminal cysteine residues (Cys17, Cys22, and Cys23). Individual substitution of each cysteine residue with serine led to a marked reduction in ARL15 S-acylation, and this reduction was more pronounced for the Cys17 and Cys22 substitutions (with the Cys23 substitution also substantially reducing S-acylation, albeit with a small residual signal), suggesting that these residues function cooperatively to facilitate efficient modification. Similar cooperative and potentially hierarchical (“priming”) relationships among nearby S-acylation sites have been proposed for other proteins, including calnexin and the calcineurin isoform CNAβ1 (26, 27). Consistent with these precedents, we speculate that S-acylation at Cys17 and/or Cys22 may act as an early and/or preferential event that promotes subsequent modification at the remaining site(s), and/or that these residues contribute disproportionately by shaping the local membrane-proximal context and accessibility of the cysteine cluster to S-acyltransferases. Although the precise mechanism underlying this cooperative S-acylation remains unknown, our observations support a model in which efficient modification of the N-terminal cysteine cluster requires coordinated contributions from multiple sites.

Protein S-acylation plays a critical role in cargo sorting and/or vesicle budding at the Golgi apparatus (28, 29). Previous studies indicate that Golgi-localized ZDHHC3 and ZDHHC7 catalyze S-acylation of a large subset of cargo proteins and promote their anterograde transport to the plasma membrane (30). Notably, ZDHHC3 and ZDHHC7 are commonly described as cis/medial Golgi-localized enzymes, supporting the idea that ARL15 S-acylation can occur in the Golgi region. Accordingly, as a nontransmembrane protein, ARL15 may be recruited to Golgi membranes where it can encounter cis/medial Golgi-resident ZDHHC3/7. S-acylation of ARL15 at this stage could promote subsequent membrane association and distribution to downstream compartments. Intriguingly, the ARL15/C23S mutant—which appears to retain S-acylation at two cysteine residues—was localized to the Golgi but not detected at the plasma membrane. This observation suggests that S-acylation at all three cysteine residues (Cys17, Cys22, and Cys23) may be required for efficient export of ARL15 from the Golgi to the plasma membrane. Alternatively, ARL15/C23S may reach the plasma membrane but exhibit reduced stability or rapid turnover at that site. Further investigations are required to elucidate the precise relationship between ARL15 S-acylation and its intracellular trafficking dynamics.

Members of the ZDHHC family primarily utilize palmitoyl-CoA (C16:0) as their lipid substrate, but they are also capable of incorporating long-chain and unsaturated acyl-CoAs (31, 32, 33). Indeed, individual ZDHHC enzymes have been shown to exhibit distinct acyl-CoA specificities (34, 35). Although ZDHHC7 and ZDHHC3 share many substrates, they differ in acyl-CoA selectivity; for example, ZDHHC7 exhibits a greater capacity to utilize stearoyl-CoA (C18:0) than ZDHHC3 (35). Thus, if both enzymes contribute to ARL15 S-acylation, it is plausible that each of the three N-terminal cysteines (Cys17, Cys22, Cys23) could carry a distinct acyl chain in *vivo*. Since the physicochemical properties of fatty acids—such as chain length and degree of unsaturation—can influence membrane affinity and the distribution of S-acylated proteins within cholesterol-rich microdomains, determining the specific acyl chains attached to ARL15 could provide deeper insights into its membrane targeting and functional roles.

Although our findings showed that ARL15 S-acylation is primarily mediated by ZDHHC7 and ZDHHC3 in HEK293T cells, we observed that a substantial fraction of S-acylated ARL15 persisted even when the activities of both enzymes were attenuated. This suggests that additional ZDHHC enzymes may also contribute to ARL15 S-acylation. Interestingly, a previous study in mouse 3T3-L1 adipocytes reported that ARL15 is S-acylated specifically at Cys22 and Cys23 and that ARL15 is released from membranes during the later stages of adipocyte differentiation (14). These observations imply that the S-acylation pattern and subcellular distribution of ARL15 may be context-dependent, potentially varying with cell type and/or culture conditions.

The physiological role(s) of ZDHHC-mediated S-acylation of ARL15 in cellular contexts remain to be fully elucidated. Given that ARL15 has been shown to form a complex with TRPM7 and CNNM family proteins, its S-acylation may influence these interactions and/or regulate the functions of associated proteins. Both TRPM7 and CNNMs are involved in intracellular Mg^2+^ homeostasis (36, 37, 38), and prior studies have suggested a potential link between ARL15 and hypomagnesemia (39). Therefore, further investigation of ARL15 S-acylation and its functional consequences may provide important insight into the regulation of Mg^2+^ balance in mammalian cells.

## Experimental procedures

### Cell culture

HEK293T cells were maintained in DMEM containing 10% FBS, penicillin, and streptomycin. The cells were seeded in polyethyleneimine (PEI)-coated dishes for each experiment (40). Stable cell lines expressing the gene of interest were generated by CRISPR/Cas9-driven targeted integration of the gene into the safe-harbor genomic locus *AAVS1*, as described previously (39). Briefly, cells were cotransfected with an expression plasmid and pCas-Guide AAVS1-T2 and selected with puromycin (0.75 μg/ml).

### siRNA transfection

Reverse transfection of siRNA was performed using Lipofectamine RNAiMAX. HEK293T cells were detached using 0.05% trypsin–EDTA (0.48 mM), resuspended in antibiotic-free medium, and seeded in 12-well plates to ∼70% confluence. siRNA and Lipofectamine RNAiMAX were separately diluted with Opti-MEM I, mixed to form complexes, and added directly to the wells containing cell suspension. After ∼24 h, the cells were detached and re-seeded into 6-well plates, followed by incubation for an additional 48 h before downstream assays. Stealth RNAi siRNA Negative Control (Med GC; Thermo Fisher Scientific, cat. 12935300) was used as a negative control. Sequences of ZDHHC-targeting siRNAs are provided in Table S1.

### Quantitative PCR

Total RNA was extracted from cultured cells using the GenElute Total RNA Purification Kit (Sigma-Aldrich) according to the manufacturer’s instructions. First-strand cDNA synthesis was performed with PrimeScript RT Master Mix (Perfect Real Time; Takara Bio). Quantitative real-time PCR was conducted using TB Green Premix Ex Taq II (Tli RNaseH Plus; Takara Bio) on a Thermal Cycler Dice Real Time System II (Takara Bio). Primer sequences are listed in Table S2. Gene expression levels were analyzed by the ΔΔCt method and normalized to GAPDH mRNA levels.

### Generation of ZDHHC7 KO cells

ZDHHC7-KO (ZDHHC7KO) cell lines were generated using the CRISPR/Cas9 system as previously described (41). HEK293T cells were transiently transfected with the pGedit-ZDHHC7 plasmid using Polyethylenimine MAX (Polysciences). At 24 h post-transfection, GFP-positive cells were isolated using a MA900 Cell Sorter (Sony). After 2 to 3 passages, single GFP-positive cells were sorted into 96-well plates for clonal expansion. Successful KO clones were validated by genomic PCR and Western blotting.

### APEGS assay

APEGS assays were performed with slight modifications from previously published protocols (17). HEK293T cells cultured in 6-well plates were washed three times with PBS and lysed in 350 μl of PBS containing 2% SDS, 8 M urea, and 5 mM EDTA. The lysates were transferred to 1.5 ml tubes and sonicated for 2 min using a Branson Sonifier 450. A 300 μl aliquot of the sonicated lysate was mixed with 6 μl of 600 mM TCEP and incubated at 37 °C for 1 h. After adding 15 μl of 1 M N-ethylmaleimide, samples were incubated for an additional 3 h at 37 °C. Proteins were precipitated by chloroform/methanol extraction and resuspended in 170 μl of PBS containing 2% SDS and 10 mM EDTA.

Samples were split into two aliquots (80 μl each) and treated with either 80 μl of PBS (*HAM*–) or 2 M neutralized hydroxylamine in PBS (*HAM*+) at 30 °C for 1 h. After another round of chloroform/methanol extraction, pellets were resuspended in 130 μl of PBS containing 2% SDS, and again split into two tubes (60 μl each). Each aliquot was mixed with 15 μl of 20 mM mPEG-2k (SUNBRIGHT ME-020MA, NOF Corporation) and incubated for 1 h at 30 °C, followed by a final chloroform/methanol extraction. The resulting pellets were resuspended in 40 μl of SDS-PAGE sample buffer (50 mM Tris-HCl pH 6.8, 10% glycerol, 2% SDS, 5% 2-mercaptoethanol, 0.02% bromophenol blue), heat-denatured at 95 °C for 5 min, and analyzed by SDS-PAGE and immunoblotting.

### Chloroform/methanol extraction

Chloroform/methanol extraction was performed as described previously (42), with minor modifications. For each 150 μl protein sample, 600 μl of methanol was added and vortexed thoroughly, followed by 150 μl of chloroform and another vortex. Subsequently, 300 μl of distilled water was added and mixed again. The mixture was centrifuged at 15,000 rpm for 3 min, and the upper aqueous layer was removed. To further wash the sample, 800 μl of distilled water was added, gently mixed, and centrifuged again. The aqueous phase was discarded, and 600 μl of methanol was added to the remaining chloroform layer, followed by vigorous mixing. After centrifugation at 15,000 rpm for 15 min, the supernatant was discarded, and the protein pellet was washed once with 600 μl of methanol. Finally, the pellet was air-dried at room temperature and resuspended in the appropriate buffer for downstream applications.

### Subcellular fractionation

All solutions used for cell fractionation were ice-cold before use. Cells cultured in a PEI-coated 10 cm dish (∼100% confluent) were washed three times with 10 ml of PBS and once with 4 ml of Buffer A (38 mM potassium aspartic acid, 38 mM potassium gluconic acid, 38 mM potassium glutamic acid, 10 mM KHCO_3_, 5 mM MgCl_2_, 10 mM triethanolamine-acetic acid (pH 7.6)). Cells were scraped off with 0.7 ml of Buffer A containing 0.5 mM AEBSF, transferred to a 1.5 ml tube on ice, and lysed by passing through a 1 ml syringe fitted with a 27G needle (15 strokes). The post-nuclear supernatant was obtained by centrifugation at 900*g*, 10 min at 4 °C and subjected to differential centrifugation at 20,000*g*, 30 min at 4 °C and 200,000*g*, 30 min at 4 °C to separate cytosolic, membrane, and microsomal fractions.

### Cell fixation and immunostaining

Cells were fixed in 4% paraformaldehyde in PBS for 15 min at room temperature and washed three times with PBS. Permeabilization and blocking were performed using a 1:1 mixture of 5% BSA in TBS (20 mM Tris-HCl, pH 7.5, 150 mM NaCl) and Blocking One (Nacalai Tesque) containing 0.1% Triton X-100 for 30 min at room temperature. After washing once with PBS, cells were incubated with primary antibodies diluted in 5% BSA/TBS for 2 h at 37 °C. Following three PBS washes, cells were incubated with fluorophore-conjugated secondary antibodies and 1 μg/ml DAPI in 5% BSA/TBS for 45 min at 37 °C. After three final PBS washes, cells were mounted using ProLong Glass Antifade Mountant (Thermo Fisher Scientific).

### Plasmids

pENTR-TiTRE-C-FLAG and pENTR-TiTRE-C-SB1 plasmids for C-terminal FLAG or SB1 tagging were generated by inverse PCR using pENTR-TiTRE as the template. Human ARL15 (NCBI accession# NM_019087.3), including WT and mutant variants, was cloned into the EcoR I/Sal I site of pENTR-TiTRE-C-FLAG. Human ZDHHC7 (NCBI accession# NM_017740.2) WT and C160S mutant were cloned into pENTR-TiTRE-C-SB1. For coexpression, ARL15-FLAG and ZDHHC7-SB1 or ZDHHC3-SB1 (NCBI accession# NM_001135179.2) were inserted into the *Eco*R I/*Sal* I and *Pac* I sites of pENTR-TRE3G-BI, respectively. LR Clonase II (Thermo Fisher Scientific) was used to recombine pENTR-TiTRE plasmids with pAAVS1_Puro_ccdb_Ubc_rtTA destination vector (43) for doxycycline-inducible expression. The pGedit-ZDHHC7 plasmid was generated by inserting the gRNA sequence targeting human ZDHHC7 (5′-GCACTTGTAGATGACTTCCC-3′) into the *Bsm*B I site of the pGedit vector (RIKEN BRC, cat. RDB16763; (41)).

### Western blotting

Proteins were resolved by SDS-PAGE and transferred to ClearTrans SP PVDF membranes (Wako) using the Trans-Blot Turbo system (Bio-Rad). Membranes were blocked with 3% skim milk in TBS containing 0.1% Tween-20 and incubated with primary antibodies. Detection was performed using EzWest LumiOne (ATTO) and imaged with the LuminoGraph II system (ATTO). Antibodies used are listed in Table S3.

### Statistical analysis

All statistical analyses were performed using JMP Pro 18 (SAS Institute; https://www.jmp.com/en/software/predictive-analytics-software). Data are presented as mean ± SD. Statistical analyses comparing multiple groups were performed using one-way ANOVA followed by Dunnett’s multiple comparisons test, and comparisons between two groups were performed using an unpaired two-tailed Student’s *t* test, as indicated in the figure legends.

## Data availability

All data generated or analyzed during this study are included in this manuscript and its supporting information.

## Supporting information

This article contains supporting information.

## Conflict of interest

The authors declare that they have no conflicts of interest with the contents of this article.
