# Supplementary material for: S-acylation and membrane localization of the small GTPase ARL15 are mediated by the Golgi S-acyltransferases ZDHHC7 and ZDHHC3
Source: J Biol Chem. 2026 Apr 16;302(6):111460. doi: 10.1016/j.jbc.2026.111460 (PMC13196370; doi:10.1016/j.jbc.2026.111460)
Supplement: Supporting information [file mmc1.pdf]

Supporting information

S-acylation and membrane localization of the small GTPase ARL15 are mediated by the Golgi S-acyltransferases ZDHHC7 and ZDHHC3

Takeshi Chino[1,*], Makoto Araki[1,*], Yuki Ashi[1], Yusuke Izawa[1], Yuji Nunami[1], Yosuke Ichikawa[1], Itsuki Kuroiwa[1], Kenji Kontani[1,‡]

[1]*Department of Biochemistry, Meiji Pharmaceutical University, 2-522-1 Noshio, Kiyose, Tokyo 204-8588, Japan*

*These authors contributed equally to this work.

[‡]Author for correspondence (kontani@my-pharm.ac.jp)

This PDF file includes:

Tables S1 to S3

Figure S1

Table S1. siRNAs used in this study

Name	Sequence
siZDHHC3_#1 (ID: s27899)	5′-CCAUGUGGUUUAUCCGUGAtt
siZDHHC3_#2 (ID: s229642)	5′-AGAAGUACUUCGUCCUGUUtt
siZDHHC5_#1 (ID: s24751)	5′-CCUCCUCAGAUGAUUCAAAtt
siZDHHC5_#2 (ID: s24752)	5′-GACACGCAAUGGAAGCCUAtt
siZDHHC7_#1 (ID: s31111)	5′-GAUGUAUUCGGAAAAUGGAtt
siZDHHC7_#2 (ID: s31109)	5′-CGAUAACUGUAAUCCUGUUtt
siZDHHC9_#1 (ID: s27467)	5′-CAAUCUAUGUCUUCGCCUUtt

Table S2. Primer sets for quantitative real-time PCR

Gene Name	Forward	Reverse
ZDHHC3	5′-AACTGTGTAGGCGAGAACAACC	5′-AGCAATGCAGGAAGTGGAATCC
ZDHHC5	5′-TATGGGTGTGTTTGGCTTTGGC	5′-AAGCCAGCCACACACATTACTG
ZDHHC7	5′-CACTCCATATGCAACGACGAGAC	5′-CAAAGACGGACTTCATCCCTTCC
ZDHHC9	5′-TGTCTTCGCCTTCAACATCGTC	5′-ACGGACCAGAGTGTAAAGAAGC
GAPDH	5′-AGCCACATCGCTCAGACAC	5′-GCCCAATACGACCAAATCC

Table S3. Antibodies used in this study

Antibodies [Working concentration or dilution]	Source	cat# Identifier
anti-DYKDDDDK (FLAG) [0.2 µg/ml]	Fujifilm Wako Pure Chemical	014-22383 RRID: AB_10659717
anti-ARL15 [0.2 µg/ml]	Abcam	ab178425
anti-Na^+/K^+-ATPase [0.2 µg/ml]	Developmental Studies Hybridoma Bank	RRID: AB_528092
anti-TGN46 [0.05 µg/ml]	BioRad	AHP500GT RRID: AB_2203291
anti-Calnexin [1:3000]	ENZO	ADI-SPA-860-D RRID: AB_2069021
anti-AKR1D1 [0.2 µg/ml]	Santa Cruz	sc-67710 RRID: AB_2224434
anti-ZDHHC7 [1 µg/ml]	Abcam	ab138210
anti-Actin [1:5000]	Fujifilm Wako Pure Chemical	016-27821
anti-SB1 [0.2 µg/ml]	Developmental Studies Hybridoma Bank	RRID: AB_579792
HRP-conjugated anti-rabbit IgG [1:20,000]	Jackson ImmunoResearch Labs	111-035-144 RRID:AB_2307391
HRP-conjugated anti-mouse IgG [1:20,000]	Jackson ImmunoResearch Labs	115-035-146 RRID:AB_2307392

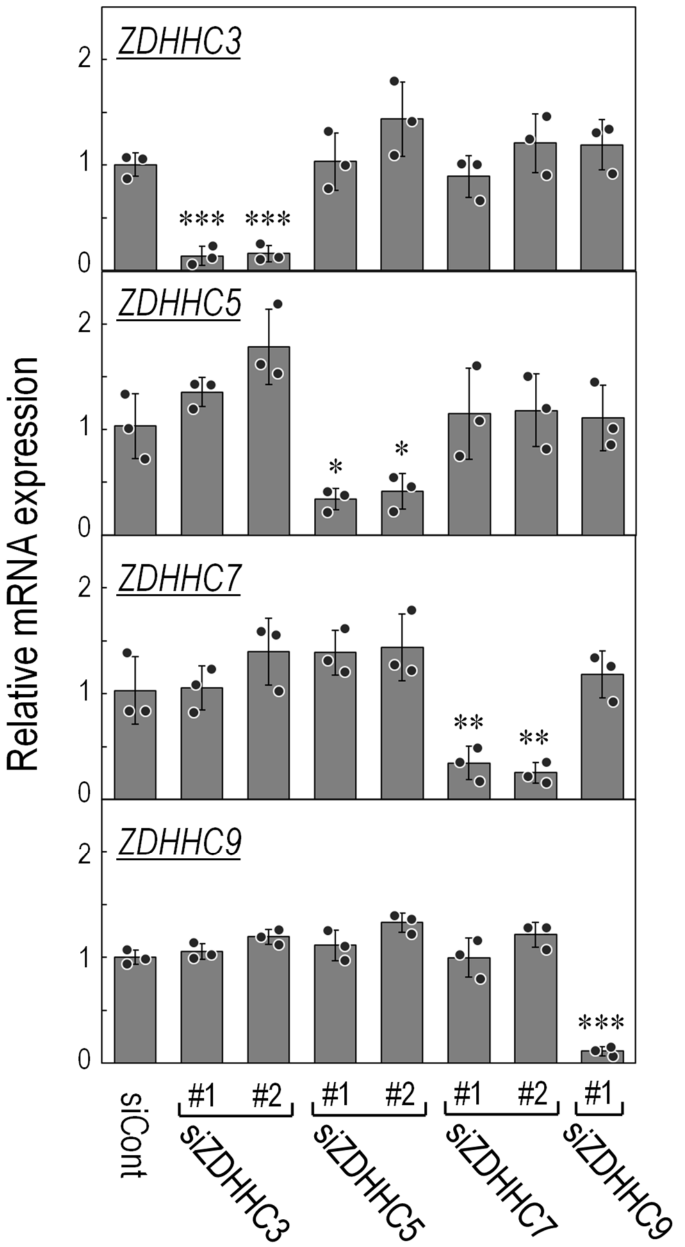

Figure S1. Quantitative (qPCR) validation of ZDHHC knockdown in HEK293T cells.

HEK293T cells were transfected with control siRNA (*siCont*) or gene-specific siRNAs targeting ZDHHC3, ZDHHC5, ZDHHC7 (two independent siRNAs per gene; labeled #1 and #2) and ZDHHC9 (single siRNA). mRNA levels were analyzed by qPCR, normalized to GAPDH, and expressed relative to *siCont* Data are means ± SD from three independent experiments, with individual data points overlaid. Statistical significance versus *siCont* was evaluated by one-sided Dunnett's multiple-comparisons test: *$p < 0.05$, **$p < 0.01$, ***$p < 0.001$.
